# Dehydro-Tocotrienol-β Counteracts Oxidative-Stress-Induced Diabetes Complications in *db/db* Mice

**DOI:** 10.3390/antiox10071070

**Published:** 2021-07-02

**Authors:** Gustav Dallner, Magnus Bentinger, Shafaat Hussain, Indranil Sinha, Jiangning Yang, Cheng Schwank-Xu, Xiaowei Zheng, Ewa Swiezewska, Kerstin Brismar, Ismael Valladolid-Acebes, Michael Tekle

**Affiliations:** 1 Rolf Luft Research Center for Diabetes and Endocrinology, Department of Molecular Medicine and Surgery, Karolinska Institutet, SE-17177 Stockholm, Sweden; Gustav.dallner@ki.se (G.D.); magnus.bentinger@sll.se (M.B.); cheng.xu@ki.se (C.S.-X.); xiaowei.zheng@ki.se (X.Z.); kerstin.brismar@ki.se (K.B.); ismael.valladolid.acebes@ki.se (I.V.-A.); 2 Department of Molecular and Clinical Medicine, University of Gothenburg, SE-41345 Gothenburg, Sweden; shafaat.hussain.turi@wlab.gu.se; 3 Department of Medicine, Division of Cardiology, Karolinska Institutet, SE-17177 Stockholm, Sweden; jiangning.yang@ki.se; 4 Childhood Cancer Research Unit, Department of Women’s and Children’s Health, Karolinska Institutet, SE-17177 Stockholm, Sweden; indranil.sinha.2@ki.se; 5 Institute of Biochemistry and Biophysics, Polish Academy of Sciences, PL-02-106 Warsaw, Poland; ewas@ibb.waw.pl; 6 Department of Clinical Pharmacology, Karolinska University Hospital, SE-17177 Stockholm, Sweden

**Keywords:** coenzyme Q, antioxidants, tocotrienol, diabetes, oxidative stress, diabetes complications, wound healing

## Abstract

Hyperglycemia, hyperlipidemia, and adiposity are the main factors that cause inflammation in type 2 diabetes due to excessive ROS production, leading to late complications. To counteract the effects of increased free radical production, we searched for a compound with effective antioxidant properties that can induce coenzyme Q biosynthesis without affecting normal cellular functions. Tocotrienols are members of the vitamin E family, well-known as efficient antioxidants that are more effective than tocopherols. Deh-T3β is a modified form of the naturally occurring tocotrienol-β. The synthesis of this compound involves the sequential modification of geranylgeraniol. In this study, we investigated the effects of this compound in different experimental models of diabetes complications. Deh-T3β was found to possess multifaceted capacities. In addition to enhanced wound healing, deh-T3β improved kidney and liver functions, reduced liver steatosis, and improved heart recovery after ischemia and insulin sensitivity in adipose tissue in a mice model of type 2 diabetes. Deh-T3β exerts these positive effects in several organs of the diabetic mice without reducing the non-fasting blood glucose levels, suggesting that both its antioxidant properties and improvement in mitochondrial function are involved, which are central to reducing diabetes complications.

## 1. Introduction

Coenzyme Q (CoQ) has been studied in detail, especially as a member of the respiratory chain in mitochondria [1]. Another main function of CoQ is participation in many cellular processes, such as antioxidant processes [1,2]. Consequently, CoQ plays important roles in maintaining cellular life and normal functions of the living organism [3]. Antioxidants have important functions in the living cell, since free radicals, such as reactive oxygen species (ROS), which are the main culprits of oxidative stress, are continuously generated and, when produced in excess, threaten normal cellular functions and biosynthetic processes [4]. Hyperglycemia, hyperlipidemia, and adiposity are the main factors that cause inflammation in type 2 diabetes due to excessive ROS production, leading to late complications [5]. To counteract the effects of excess free radical production in living organisms, we searched for a compound that has effective antioxidant properties and can induce CoQ biosynthesis without affecting normal cellular functions.

**Figure 1:**
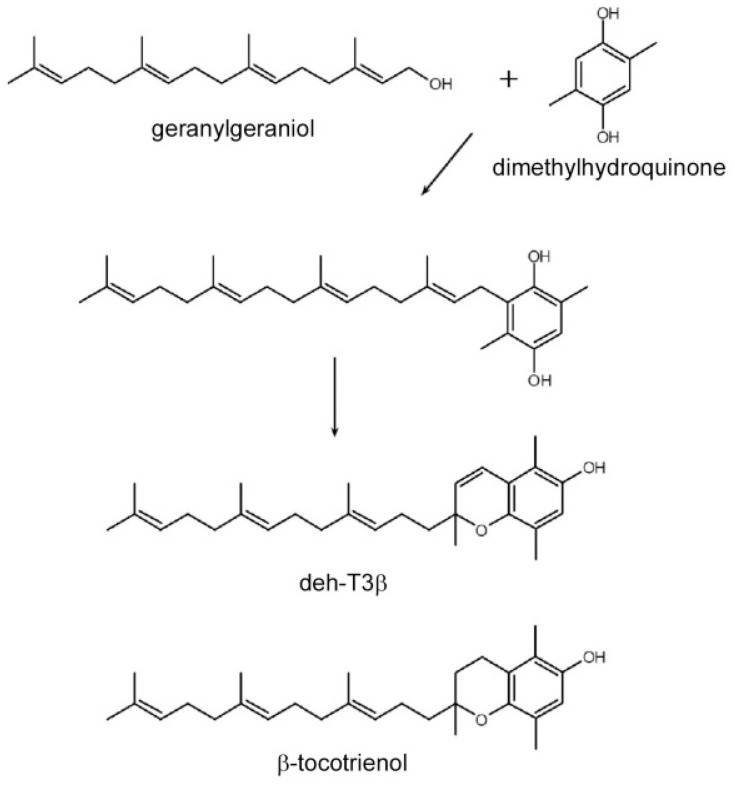
Structure and synthesis of dehydro-tocotrienol-β (deh-T3β). For comparison, the structure of the naturally occurring β-tocotrienol is shown.

Tocotrienols are members of the vitamin E family, well-known as efficient antioxidants that are more effective than tocopherols [6]. Tocotrienols have three unsaturated isoprene units in the sidechains, whereas the side chains of tocopherols are saturated. Chemically epoxidated all-trans-polyprenols induced CoQ biosynthesis in HepG2 cells [7]. One of the epoxidated tocotrienol derivatives, mono-epoxy-tocotrienol-α (MeT3α), enhanced in vivo wound healing in *db/db* mice, in vitro wound healing performed on human dermal fibroblast cells, and in vitro angiogenesis in human dermal microvascular endothelial cells (HDMVEC) [8]. Encouraged by the diverse positive effects of MeT3α, we wanted to explore the effects of different modifications of the tocotrienol compounds. We thus synthesized many various derivatives of the tocotrienol family. The experiments that were set as standards for selecting the best products included in vivo and in vitro wound healing in fibroblasts, in vitro angiogenesis in HDMVEC, mRNA expression of the growth factors *PDGFB* and *VEGFA* in fibroblasts, as well as stimulation of CoQ biosynthesis in HepG2 cells. We found that the best effects in the experimental systems were achieved with one of the modified derivatives, dehydro-tocotrienol-β (deh-T3β), even compared to the naturally occurring tocotrienols α, β, γ, and δ. Deh-T3β is a modified form of the naturally occurring tocotrienol-β. The synthesis of this compound involves the sequential modification of geranylgeraniol (Figure 1).

It appeared of considerable interest to extend our studies on the efficiency of this compound to different experimental models of diabetes complications. Our findings show that deh-T3β enhances both in vitro and in vivo wound healing, induces the formation of new capillary blood vessels in in vitro systems, and increases mRNA expression of growth factors *PDGFB* and *VEGFA*. Furthermore, it improves kidney function, liver function by reducing liver steatosis, heart recovery after ischemic reperfusion injury, and insulin sensitivity in adipose tissue in a mice model of type 2 diabetes.

## 2. Materials and Methods

### 2.1. Synthesis of Deh-T3β

Synthesis of the mixed stereoisomers (2S) and (2R) of dehydro-tocotrienol beta (deh-T3β) was performed according to the procedure by Chandrasekhar et al. [9] with minor modifications. We mixed 7 g of 2,5-dimethyl-1,4-benzoquinone in 25 mL ethanol and 35 mL diethyl ether. The benzoquinone ring structure was then reduced by dissolving in 0.3 g NaBH_4_. The reduced product was dissolved in 25 mL water free 1,4-dioxane and 15 mL geranyl-geraniol by heating to 50 °C in an oven while simultaneously mixing. We added 5 mL fresh boron trifluoride diethyl etherate (mixed with 7 mL 1,4 dioxane) in portions under rigorous mixing and incubated for 1–2 h. The reaction was followed on TLC, and more trifluoride diethyl etherate was added to accelerate the reaction. The product was extracted by adding 35 mL diethyl ether and 100 mL 1 M NaCl. The ether phase was washed twice with 100 mL 1 M NaCl. The water phase was collected in 15 mL of diethyl ether to recover some of the lost product. The organic phases were then pooled in a 50 mL glass tube, and the solvent was evaporated by nitrogen. Finally, the product was dissolved in 25 mL hexane. To oxidize the ring structure, 10 g of AgO was added to the mixture and mixed for 10 min. The solution was then transferred to a new tube and the remaining AgO was washed twice with 2 mL hexane. The pooled solution was then placed on a silica column. The product was eluted starting with 100% hexane, increasing to a hexane:diethyl ether ratio of 95:5. The oxidized products that were identified by TLC were pooled together and evaporated under a stream of nitrogen.

#### 2.1.1. Ring Closure

The products were dissolved in 15 mL pyridine and incubated in an oven for 75 min at 150 °C. The mixture was left to cool, and the compound was extracted with 50 mL 0.5 M NaCl and 25 mL diethyl ether. The remaining pyridine was washed from the ether phase 4 times with 0.5 M NaCl. 

#### 2.1.2. Purification of Final Product

After the evaporation of ether, the sample was dissolved in hexane and placed on a silica column. It was finally eluted with a gradient from 100% hexane to 80:20 hexane:diethylether. The tubes containing the final products were separated with TLC and the solvent was evaporated under nitrogen. The appropriate molecular weight of the final product (408.6 g/mol) was established with mass spectrometry. We used [^3^H] sodium borohydride (10 Ci/mmol; Amersham Pharmacia Biotech, UK) to label the chromanol structure of deh-T3β according to Keenan and Kruczek [10]. The final product, deh-T3β, was synthesized so that the metabolites produced retained radioactivity according to Bentinger et al. [11].

### 2.2. Cell Culturing and qPCR Gene Expression Analysis

Human dermal fibroblast (HDF) cells were purchased from American Type Culture Collection (ATCC, Manassas, VA, USA). HDF cells were grown in Dulbecco’s modified Eagle’s medium (Invitrogen, Waltham, MA, USA) containing 5 mM glucose, supplemented with 10% fetal bovine serum, penicillin (100 units/mL), and streptomycin (100 mg/mL). Then, 0.1–0.5 μg of total RNA was reverse-transcribed into cDNA using random hexamer oligonucleotides and TaqMan reverse transcription reagents (Applied Biosystems, Foster City, CA, USA) in a final volume of 20 μL. The cDNA was mixed with SYBR Green PCR master mix and specific primers. PCR analysis was performed with an ABI Prism 7300 sequence detector (Applied Biosystems).

### 2.3. Microarray Gene Expression Analysis in Deh-T3β-Treated Mice Liver

The Affymetrix U133 plus 2.0 GeneChips Human array covering 54,000 genes was used for gene expression analysis. Samples were analyzed at the Department of Oncology-Pathology (OnkPat), K7, Karolinska University Hospital, in accordance with the manufacturer’s instructions (Affymetrix, Regnsburg, Germany). *Db/db* mice were intraperitoneally injected with 1 μmol deh-T3β every other day for three weeks and controls were treated with ethanol 10 mL/kg body weight. RNA was isolated using the QIAGEN Mini RNeasy Plus Minikit according to the manufacturer’s instructions. Prior to array, RNA integrity was checked using an Agilent Bioanalyzer 2100 (Agilent Technologies, Santa Clara, CA, USA). Complementary DNA (cDNA) synthesized from total RNA was used for synthesis and isolation of biotin-labeled complementary RNA (cRNA) and fragmented to a mean size of ~50–100 nucleotides. Triplicate samples were used for each experimental condition and the raw data files were analyzed by Affymetrix Expression Console followed by Qlucore Omics Explorer. The robust multiarray averaging (RMA) normalization method was applied, and fold-change values were calculated. Functional pathway analysis was performed using ingenuity pathway analysis software (IPA). 

### 2.4. Extraction of Labeled Deh-T3β Metabolites in Mice Organs

To evaluate the tissue distribution of deh-T3β and its metabolites, *db/db* mice were intraperitoneally injected with 0.5 µCi ^3^H-labelled deh-T3β and another five were injected with the vehicle alone (10 mL/kg body weight of ethanol). For dietary studies, equal numbers of mice were used (five controls and five treated). Then, ^3^H-labelled deh-T3β (1 mmol/kg chow) was impregnated into the chows and the mice had free access for 10 days. The mice were sacrificed after 24 h and after 10 days, respectively, and organs and blood were isolated. Tissues were homogenized in 150 mM NaCl. Metabolites were then extracted in 1 mL chloroform/methanol (1:1) at 37 °C with magnetic stirring for 1 h. Extracts were adjusted to a final chloroform/methanol/water ratio of 3:2:1, and phase separation was accomplished by centrifugation [11]. An aliquot of the methanol/water phase and chloroform phase was mixed with scintillation liquid and radioactivity was detected by a liquid scintillation counter. 

### 2.5. Animals and Experimental Protocol

Animal studies were approved by the North Stockholm Animal Ethics Committee No: N199/13. Male C57BL6 wild-type (wt) and *db/db* micewere purchased from Charles River Belgium at 12 weeks of age. Animals were housed five animals per cage in a 12 h light/12 h dark cycle at 22 °C and provided with standard chow and water ad libitum. The animals were acclimatized for one week before the experiments. Five male wt and five *db/db* mice 13 weeks of age were used for the different experiments. Deh-T3β was dissolved in ethanol and mixed with chow (1 g deh-T3β per kg chow) and the animals were fed for two weeks to two months ad libitum. We studied the effect on wound healing, red blood cells, and on the heart, kidney, and liver function.

### 2.6. Wound Healing

For wound-healing purposes, ten C57BL6/KsJm/ *Leptdb* (*db/db*) animals, 13 weeks of age, were held individually for one week and were cared for daily in a 12 h light/12 h dark cycle at 22 °C and provided *ad libitum* with standard chow and water. The mice (n = 5) were wounded by two 6 mm punch biopsies on the dorsum, one on each side of the midline, as described previously [12]. Both animal groups were treated every other day locally with a suspension of 1 µmol deh-T3β or vehicle (PEG dissolved in PBS) according to the method previously described [8]. The animals were caged separately during the healing process with one animal per cage and the wounds were treated every other day until 90% wound closure or for 15 days. The mice were then sacrificed by decapitation. All the studies on the mice were approved by the North Stockholm Animal Ethics Committee.

### 2.7. Determination of p66Shc, and p47-phox by Immunoblotting in Mice Heart

Organs from C57BL6 (wt) and *db/db* mice were isolated and snap-frozen in liquid nitrogen. Frozen samples were successively pulverized and dissolved in lysis buffer containing 50 mM Tris-HCL (pH 7.4), 100 mM NaF, 2 mM EDTA, 1 mM DTT, 2 mM NaVanadate, 8.5% sucrose, 5 μg/mL aprotinin, 100 μg/mL leupeptin, and 5 μg/mL pepstatin. Protein concentrations were determined using Bio-Rad Protein Assay Kits according to the manufacturer’s protocol. Proteins were loaded on a separating denaturing SDS/10% polyacrylamide gel. Membranes were incubated with primary anti-p66Shc (Abcam), Mouse anti-p47phox antibody was purchased from Santa Cruz Biotechnology (Dallas, TX, USA). Primary anti-GAPDH (Millipore Corporation, Billerica, MA, USA) antibodies were used as the loading control. Protein expressions were detected by enhanced chemiluminescence System (Millipore, Bellerica, MA, USA). Densitometric quantification of protein bands was analyzed using ImageJ software (National Institutes of Health, Bethesda, MD, USA).

### 2.8. Preparation of Red Blood Cells (RBCs) and Heart Perfusion

Mouse whole blood was collected from the thoracic cavity after removal of the heart used in the experiment. RBCs were obtained by removing the plasma and buffy coat including top part of the RBC layer after centrifugation at 1000× *g* at 4 °C for 10 min. The final RBC suspension was achieved by washing 3 times in Krebs Henseleit (KH) buffer and 1:1 dilution with KH buffer [13].

Following anesthesia, hearts were isolated from wt C57BL6 mice and perfused in a Langendorff system [14]. After stabilization, the hearts were subjected to 40 min global ischemia followed by 60 min reperfusion. Red blood cells (RBCs) from deh-T3β-treated and non-treated *db/db* mice were administered to isolated wt mouse hearts according to Yang et al. [13]. The method allows the RBC suspension to be present in the heart during the ischemic period and was washed away by the reperfusion.

#### Detection of Reactive Oxygen Species in Red Blood Cells from db/db Mice

To evaluate the antioxidant capacity of deh-T3β, ROS levels in RBCs were determined using electron spin resonance (ESR). Washed RBCs from *db/db* mice were diluted to 1% hematocrit with krebs/N-2-hydroxyethylpiperazine-N-2-ethanesulfonic acid buffer. The RBCs from deh-T3β- (1 mmol/L) or vehicle (ethanol)-treated mice were assayed for the presence of ROS, which was detected by electron spin resonance according to Yang et al. [13].

### 2.9. Tissue Fixation for Histological Studies of the Kidney

For tissue fixation, mice were anesthetized with isoflurane, and transcardially perfused with PBS followed by freshly prepared 4% paraformaldehyde in PBS (pH 7.4). Kidneys were dissected out and post-fixed overnight. After fixation, tissue samples were processed with a sucrose gradient (10–30% (*w*/*v*) sucrose solution in PBS containing 0.01% (*w*/*v*) sodium azide and 0.02% (*w*/*v*) bacitracin), frozen in dry ice and preserved at −80 °C until use.

### 2.10. Hematoxylin and Eosin Staining of Mouse Kidney

Paraformaldehyde-fixed kidneys were sectioned into 20 μm thick longitudinal sections with a cryostat (Microm HM500M/Cryostar NX70; Thermo Scientific, Stockholm, Sweden) and collected onto SuperFrost Plus microscope slides (VWR International, Stockholm, Sweden). After 2 h equilibrating at room temperature, tissue sections were rinsed in hematoxylin (Sigma-Aldrich, Stockholm, Sweden; 50% vol/vol diluted in distilled water) for 6 min, washed with distilled water, and stained with eosin (Histolab; Gothenburg, Sweden) for 30 s. After washing, sections were dried and mounted with VectaMount permanent mounting medium (Vector Laboratories, Burlingame, CA, USA). Once the mounting medium was solidified, the preparations were imaged under a 10× magnification objective using an optical microscope (Leica, Stockholm, Sweden). The morphological evaluations were performed in three non-consecutive sections separated by 100 µm. For each section, 3 fields of view were collected randomly in 3 to 4 individual animals per experimental group.

### 2.11. Spectrophotometric Analysis of Liver Triglycerides

Liver tissues were dissected out, weighed, snap-frozen in liquid nitrogen, and stored at −80 °C until use. Hepatic triglyceride content was determined, as previously described [15], by carcass saponification in 0.1 M KOH in 99% ethanol. Triglycerides were analyzed using Free Glycerol Reagent and Glycerol Standards (Sigma-Aldrich, Darmstadt, Germany) to construct the standard curve. The glycerol concentration (triolein equivalents) was measured by spectrophotometry (SAFAS-MONACO) at λ = 540 nm and determined by extrapolation from the standard curve. Total triglycerides content is expressed as mg per g of liver tissue.

### 2.12. Determination of Triglycerides, Creatinine, Leptin, and Adiponectin Levels in the Blood

Blood glucose was determined using a FreeStyle Glucometer (Abbot Diabetes Care, Alameda, CA, USA). Blood triglycerides levels were determined using a multi-parameter diagnostic device (multiCare-in, Biochemical Systems International S.r.l., Arezzo, Italy). Plasma creatinine levels were measured in deproteinized plasma by centrifugation in 10 kDa spin columns (Abcam, Cambridge, UK) using a commercial colorimetric kit (Abcam, Cambridge, UK). Plasma levels of aspartate transaminase (AST) and alanine aminotransferase (ALT) were routinely determined in the clinical laboratory. Plasma leptin and adiponectin were measured using ELISA (Phoenix Pharmaceuticals Inc., Burlingame, CA, USA, for leptin; CrystalChem, Downers Grove, IL, USA, for adiponectin).

### 2.13. Determination of Mitochondrial Swelling

Mitochondrial swelling was assessed by measuring the absorbance of their suspension at 540 nm. Kidney mitochondria of deh-T3β treated and non-treated *db/db* mice were prepared in assay buffer (1.0 mg protein/mL) containing 125 mmol/L sucrose, 50 mmol/L KCl, 2 mmol/L KH2PO4, 5 μmol/L rotenone, 10 mmol/L Hepes, and 5 mmol/L succinate. The extent of mitochondrial swelling was assayed by measuring the decrease in absorbance (A540) every 30 s for 7 min with a multiplate reader (MD) after the addition of 50 μmol/L Ca^2+^ at 30 °C [16].

### 2.14. Mitochondrial Function and Oxygen Consumption Rate Measurement by Seahorse XF-24

H9C2 rat myoblast cells (ATCC, Manassas, VA, USA) were grown in Dulbecco’s modified eagle’s medium (DMEM) modified to contain 4 mM L-glutamine, 4500 mg/L glucose, 1 mM sodium pyruvate, and 1500 mg/L sodium bicarbonate supplemented with fetal bovine serum to a final concentration of 10%. Treatment with deh-T3β was performed at cell passages 5–7. Prior to analysis, cells were trypsinized and seeded in 24-well Seahorse XF-24 (Seahorse Bioscience, Billerica, MA, USA) plates at a density of 30,000 cells per well in standard growth medium and incubated at 37 °C overnight. On the second day, cells were washed twice with 400 μL unbuffered XF-assay medium containing 1 mM pyruvate, 1% FBS, and 2 mM glutamine, adjusted to pH 7.4, and incubated in a 0% CO_2_ incubator at 37 °C for 3 h in the presence of EtOH (5%), deh-T3β (10 nmol), H_2_O_2_ (0.5%), and deh-T3β+H_2_O_2_. The assay protocol consisted of 3 min mix, 2 min waiting and 3 min measurement cycles. Three readings of oxygen consumption rate (OCR) were recorded after each addition of mitochondrial inhibitor before injection of the subsequent inhibitors. The mitochondrial inhibitors used were the ATP synthase inhibitor oligomycin (1 μM), the uncoupler ionophore carbonylcyanide p-trifluoromethoxyphenylhydrazone (FCCP; 1 μM), and the complex III inhibitor antimycin A (1 μM). The basal respiration, ATP turnover rate, proton leak, and maximal and spare respiratory capacity of the mitochondria were determined using these inhibitors [8,17].

## 3. Results

### 3.1. Synthesis of Dehydro-T3β (Deh-T3β)

Deh-T3β was synthesized as described in the Materials and Methods section. The product, deh-T3β, was dissolved in absolute ethanol stored in a −20 °C freezer and was stable for 12 months. There was no degradation of the product during this period.

### 3.2. Gene Expression Analyses

**Figure 2:**
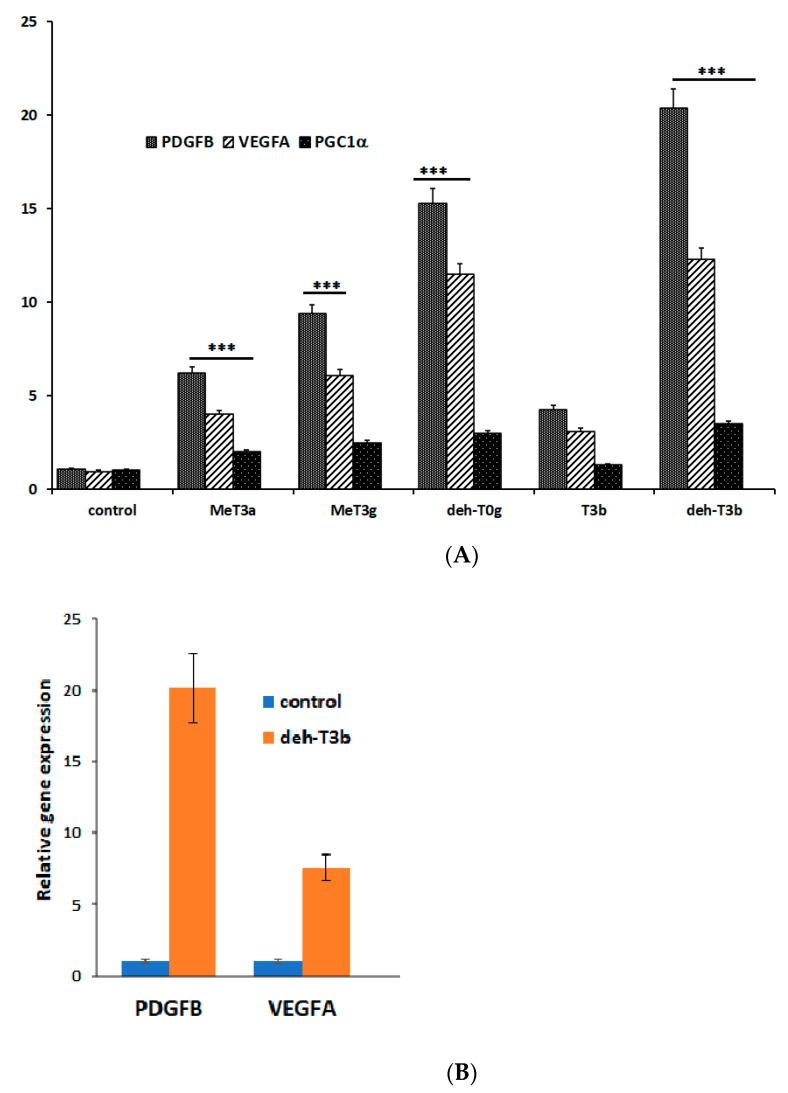
(**A**) Overnight serum-starved human dermal fibroblast (HDF) cells were cultured in the presence of 10 μM of each of the modified tocotrienol products for 3 h. Total RNA was isolated and gene expression was analyzed by qPCR (ABI 7300, Applied Biosystems). The values provided are the mean ± SD (n = 5). Control, HDF cells incubated in the presence of vehicle (ethanol); MeT3α, mono-epoxy-tocotrienol-α (naturally occurring tocotrienol-α with one epoxide on its side chain described in [8]); MeT3α, mono-epoxy-tocotrienol-α (naturally occurring tocotrienol-α with one epoxide on its side chain); deh-T0γ, dehydro-tocotrienol-γ (naturally occurring tocotrienol-γ with truncated side-chain); T3β, tocotrienol-β (naturally occurring tocotrienol-β); deh-T3β, naturally occurring tocotrienol-β with a double bond on the chromanol ring as described in Figure 1. All these products upregulate the expression of growth factors *PDGFB* and *VEGFA* as well as *PGC1α*. Data are normalized to the expression of *HMBS1* in each sample and presented as the relative expression of the control group. Values are mean ± SD; *** *p* < 0.001. (**B**) Gene expression levels of *PDGFB* and *VEGFA* in skin from mice treated with deh-T3β.

HDF cells were treated with several modified tocotrienols to investigate their effects on the mRNA expression of growth factors such as *PDGFB* and *VEGFA* and deh-T3β was found to be the most efficient product, as shown in Figure 2A, and in mice skin (Figure 2B).

### 3.3. Microarray Gene Expression Analysis in Deh-T3β-Treated Mice Liver

**Figure 3:**
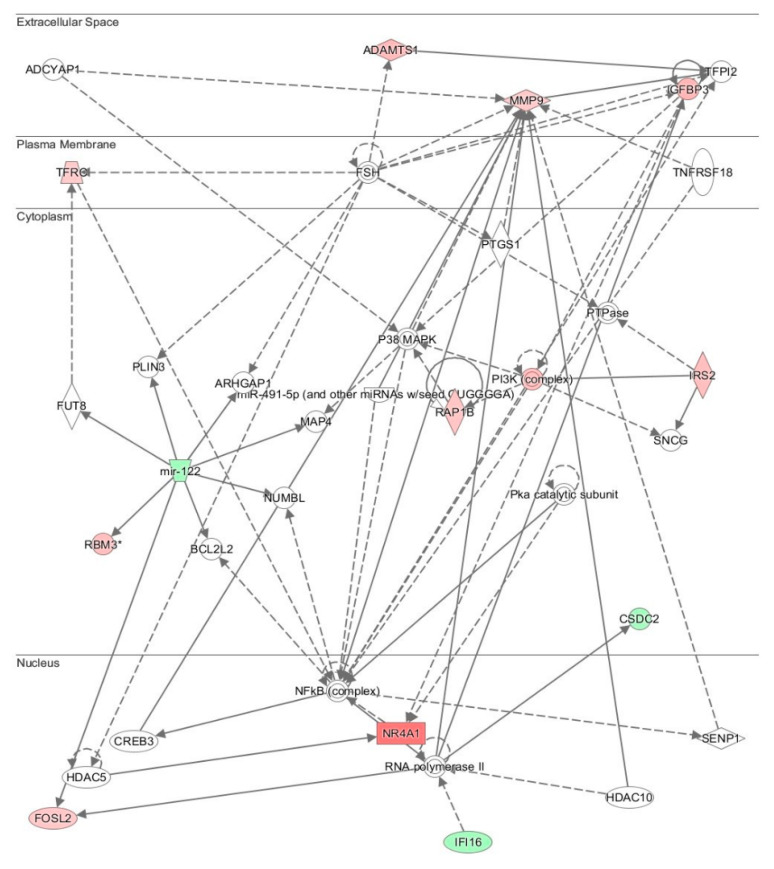
Ingenuity Systems Pathway (IPA) analyses. Network of genes affected by deh-T3β treatment. Upregulated genes are shown in red shaded shapes, and downregulated genes are shown in labeled green shaded shapes. Non-colored areas represent genes added by IPA when searching for biological relationships using *NR4A1* as the searching center.

Microarray analysis after three weeks of treatment showed that the gene expression fold-increase ranged from 4.7-fold of the highest to a 1.2-fold increase in 148 genes. Nuclear receptor (*NR4A1)*, which is involved in the regulation of multiple cellular processes such as metabolism, proliferation, and migration, showed a 3.2-fold gene expression increase, while 208 genes were downregulated by 4.4-fold to 0.8-fold. The lowest decrease was observed in *MIR669b*. A gene network search by Ingenuity Pathway Analysis (IPA) software directed around the NR4A1 gene was performed. Three genes whose translated products are involved in a number of cellular functions were identified as having a direct or indirect association with the genes that are upregulated (red shaded structures) and downregulated (green shaded shapes) in Figure 3. The genes include *PI3K*, *P38-MAPK*, and *NF-KB*.

### 3.4. Distribution of Labeled Deh-T3β Metabolites in db/db Mice Organs

**Figure 4:**
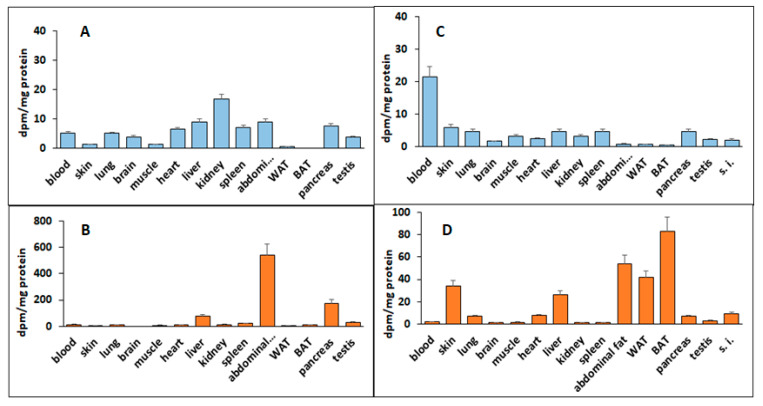
Deh-T3β metabolism and metabolite distribution in different organs of *db/db* mice (n = 3). (**A**) Hydrophilic phase (methanol:water) 24 h after treatment by intraperitoneal injection; (**B**) hydrophobic phase (chloroform) 24 h after intraperitoneal injection of deh-T3β; (**C**) hydrophilic phase after 10 days of deh-T3β treatment by diet; (**D**) hydrophobic phase after 10 days’ deh-T3β treatment by diet. Results are means ± SD of three independent experiments.

We intraperitoneally injected *db/db* mice (n = 3) with tritium-labeled deh-T3β. The animals were sacrificed after 24 h and the distribution of the labeled metabolites were analyzed in different tissues. In the water/methanol phase, metabolites were distributed in all organs, but mainly in the liver, kidney, spleen, and abdominal fat (Figure 4A). In the organic phase, the radioactive compound was mainly accumulated in the liver, abdominal fat, and pancreas (Figure 4B).

When the same compound was administered to the animals (n = 3) orally for ten days, distribution of the radioactive metabolites showed a different pattern. In the water phase, the metabolite was present in all organs analyzed and was mostly present in the blood (Figure 4C). In the organic phase, the fat-soluble non-metabolized compound (deh-T3β) was also present in all the organs, but highly accumulated in the skin, liver, abdominal fat, and white and brown adipose tissues (Figure 4D).

### 3.5. In Vivo Wound Healing

**Figure 5:**
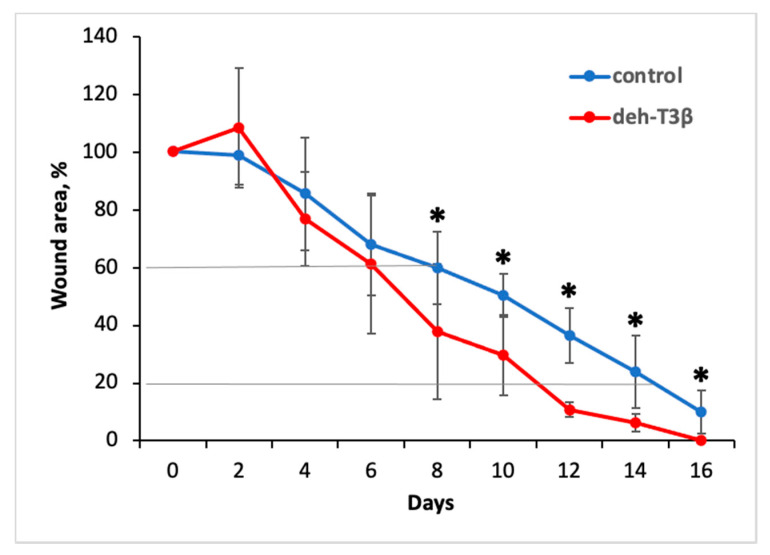
Effect of local treatment with deh-T3β on wound healing in diabetic *db/db* mice. The healing rate of full-thickness wounds in *db/db* mice was promoted by local treatment with 1 μmol deh-T3β compared to the vehicle (values are means ± SEM (* *p* < 0.05, *db/db*-treated vs. *db/db* placebo)). Diagonal lines denote 40% healing rate (upper line) after six and eight days for the treated and controls, respectively; and 80% healing rate (lower line) after eleven and fifteen days for treated and controls, respectively.

The wound healing process in diabetes is impaired due to hyperglycemia induced oxidative stress and reduced activity of HIF-1α, followed by decreased neovascularization and tissue formation [1]. In this study we treated the wounds locally with deh-T3β or vehicle. The deh-T3β-treated *db/db* mice showed significantly faster wound healing compared to the non-treated *db/db* control mice, and the differences in the mean wound areas were statistically significant at all time points after day 8 (Figure 5). We observed 80% wound closure after 11 days in the *db/db* mice treated with deh-T3β compared to 14 days in those treated with vehicle. We observed 90% closure of the wound area in the deh-T3β-treated diabetic mice, four days earlier than in those treated with vehicle.

### 3.6. Antioxidant Function of Deh-T3β in Mice Red Blood Cells

**Figure 6:**
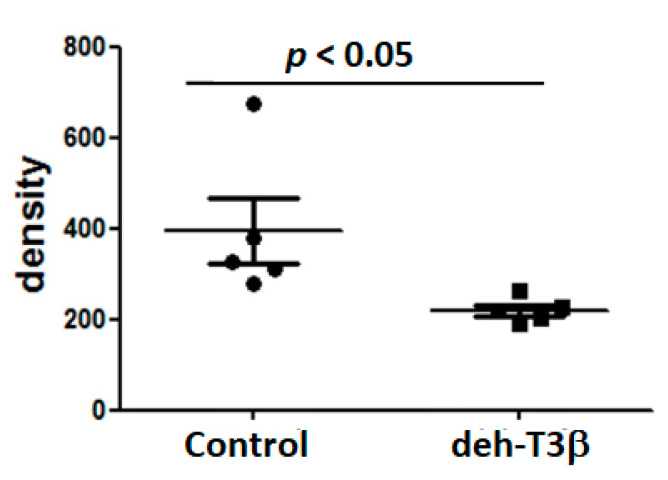
Antioxidant capacity of deh-T3β in red blood cells from non-treated *db/db* mice (control) and red blood cells from mice treated with deh-T3β for one month by diet.

The antioxidant capacity of deh-T3β was investigated in the red blood cells of *db/db* mice treated with deh-T3β compared to red blood cells from non-treated *db/db* mice using electron spin resonance (ESR). In the non-treated mice, the ROS levels in the red blood cells were high (Figure 6), but the ROS levels were significantly lowered by 50% (*p* < 0.05) after one month of treatment with deh-T3β in the diet.

### 3.7. Expression of p66 in the db/db Mice Heart Treated Orally with Deh-T3β

**Figure 7:**
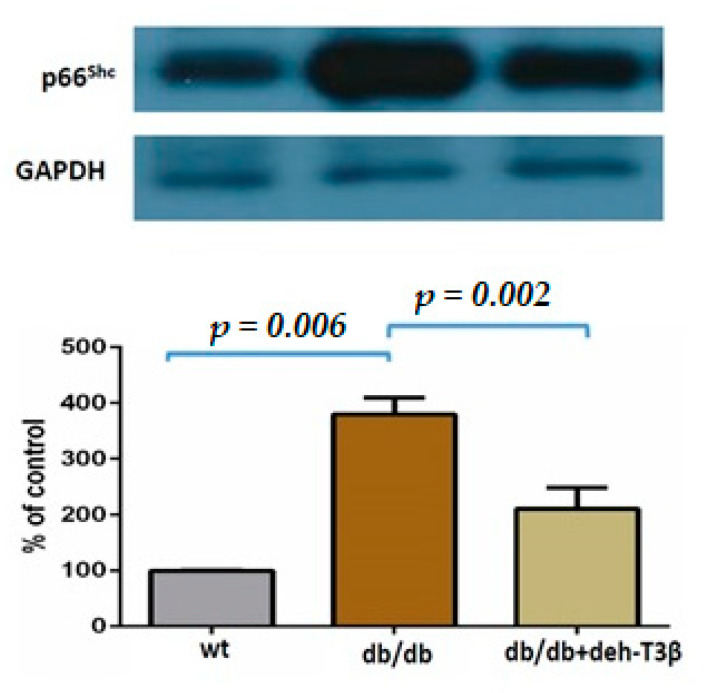
Western blot analysis of p66Shc expression in wt and *db/db* mice heart. *db/db* mice were fed with deh-T3β mixed with the diet for two months.

The mitochondrial adaptor p66Shc is an important molecular effector involved in metabolic and cardiovascular diseases. This enzyme plays a major role in the generation of reactive oxygen species [18]. Phosphorylation of p66Shc at Ser-36 induces the transfer of the protein from the cytosol to the mitochondrion, where it increases ROS accumulation [19]. This latter event leads to mitochondrial disruption and subsequent apoptosis. Western blot analysis demonstrated a significant increase in non-treated *db/db* mice and a decrease (*p* = 0.002) of this protein after dietary treatment for two months with deh-T3β compared with the non-treated *db/db* mice (Figure 7).

### 3.8. Expression of P47-Phox in db/db Mice Heart Treated Orally with Deh-T3β

**Figure 8:**
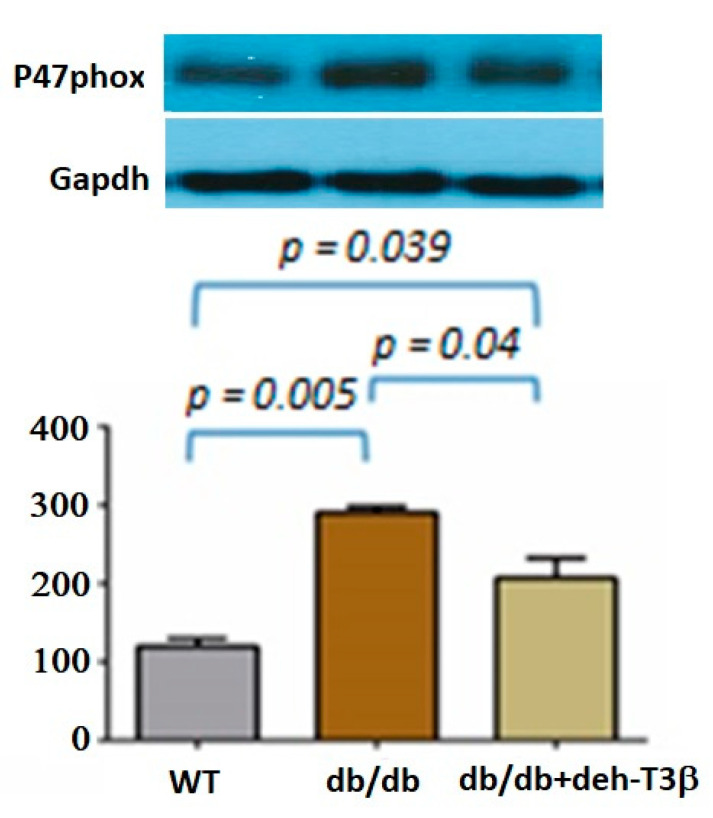
Western blot analysis of P47-phox expression in *wt and db/db* mice heart. *Db/db* mice were fed with deh-T3β mixed with the diet for two months.

P47-phox, the phagocyte NADPH-oxidase is involved in the generation of superoxide anion and other ROS molecules [20]. The expression of this protein in *db/db* mice heart was very high compared to the wt mice. In the hearts of *db/db* mice treated for two months with deh-T3β, in the diet, the expression of this enzyme was decreased (*p* = 0.04; Figure 8).

### 3.9. Effect of Deh-T3β on Heart Recovery after Ischemia Reperfusion Injury

**Figure 9:**
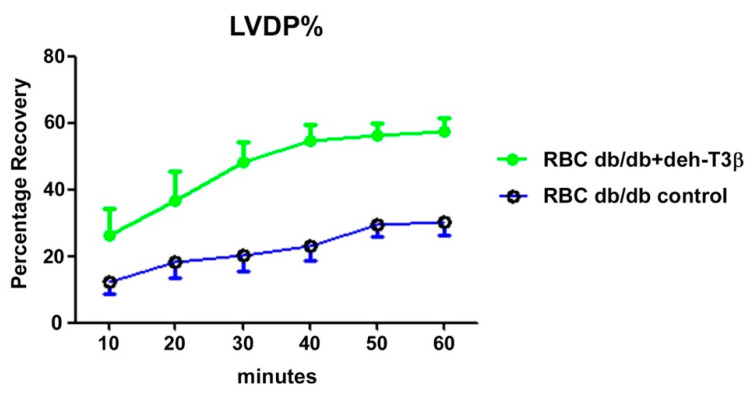
*db/db* mice were fed with deh-T3β mixed in the diet or standard chow (for the control group) for one month. Blood from these mice was used to study the non-diabetic C57B6 mouse heart response to ischemic reperfusion injury for 40 min.

Heart recovery determined as Left ventricular developed pressure (LVDP) after ischemia-reperfusion injury was analyzed by a Langendorff system where isolated hearts from healthy C57BL6 control mice were employed. In this system, the heart was subjected to global ischemia for 40 min [13]. Red blood cells were added to the perfusion from either deh-T3B-treated or non-treated *db/db* mice before measuring the recovery of the heart from the ischemic reperfusion injury. A 60% recovery was registered when red blood cells from the deh-T3β treated *db/db* mice were employed for perfusion, whereas there was only 30% recovery when red blood cells from non-treated *db/db* mice were used (Figure 9).

### 3.10. Antioxidant Effect of Deh-T3β in Rat Cardiac Myoblast Cells (H9C2)

**Figure 10:**
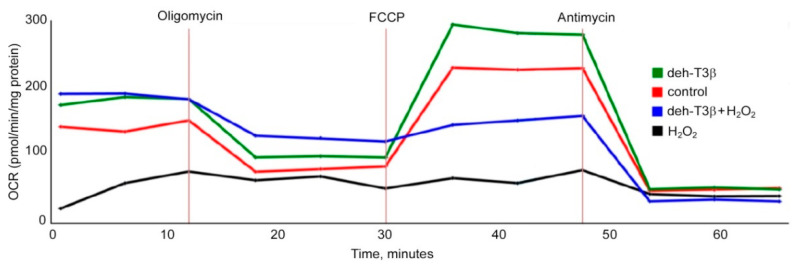
Seahorse respiratory assay in H9C2 cells incubated with deh-T3β in the presence of H_2_O_2_.

H9C2 cells were treated with deh-T3β in the presence of H_2_O_2_. The deleterious effect of H_2_O_2_ decreased, and deh-T3β partially restored the respiratory capacity of the cells. Remarkably, the effect in the presence of deh-T3β exceeded the respiratory capacity of the controls (Figure 10).

### 3.11. Morphological Appearance of the Kidney after Four Weeks of Oral Treatment with Deh-T3β

**Figure 11:**
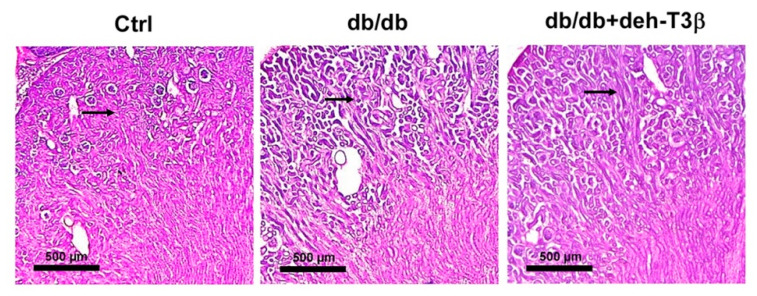
Histology of H&E-stained mice kidney sections. Ctrl wt C57Bl6 kidney, kidneys from non-treated *db/db,* and deh-T3β treated *db/db* mice for four weeks ad libitum.

In normal healthy kidneys, the glomerular and tubular structures are well-organized and show no pathological signs. In contrast, *db/db* mice kidneys were characterized by degeneration and fat accumulation in both the glomerular and tubular structures. After treatment with deh-T3β for four weeks, however, the kidney showed a substantial recovery of both glomerular and tubular structures, as depicted in Figure 11 and denoted by the arrows.

### 3.12. Mitochondrial Swelling

**Figure 12:**
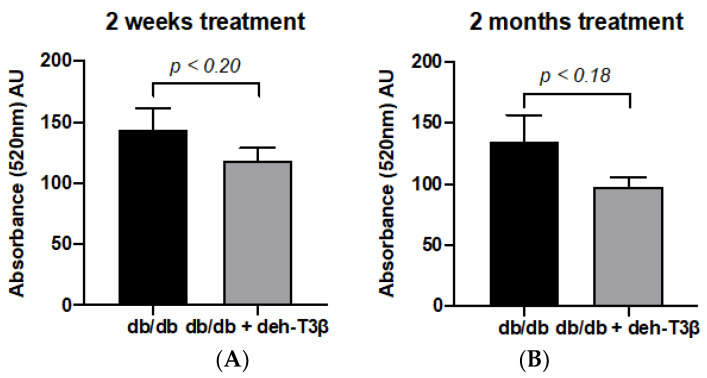
Mitochondrial swelling in kidneys of *db/db* mice. Each column represents mean values ± SD. *db/db* control mice (n = 5) compared to deh-T3β-treated mice (n = 5) after (**A**) 2 weeks’ and (**B**) 2 months’ treatment. *p* < 0.18.

Mitochondrial swelling of both treated and non-treated *db/db* mice was measured in kidney mitochondria after two weeks and two months. Mitochondria from non-treated mice showed increased swelling (more leakage), which was partially restored (*p* < 0.18) after both two weeks’ and two months’ treatment with deh-T3β (Figure 12A,B).

### 3.13. Creatinine Levels in Mice Plasma

**Figure 13:**
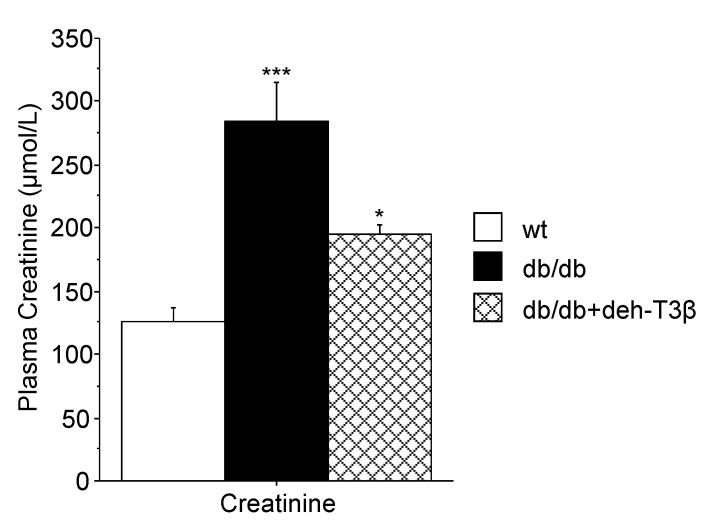
Creatinine levels in plasma of wt C57BL6 mice and of *db/db* mice, non-treated and treated with deh-T3β for two weeks ad libitum. Values are means ± SEM of three independent experiments, * *p* < 0.01; *** *p* < 0.001.

Creatinine levels in plasma were analyzed in wt (C57BL6 mice) and in deh-T3β-treated and non-treated *db/db* mice (n = 5/group). The creatinine levels in the *db/db* mice were at least twice as high as those of normal wt mice, indicating kidney dysfunction. When treated with deh-T3β, the creatinine levels of the *db/db* mice decreased significantly (*p* < 0.01) showing a partial recovery of kidney function (Figure 13).

### 3.14. Appearance of the Liver in Non-Treated and Deh-T3β Treated db/db Mice

**Figure 14:**
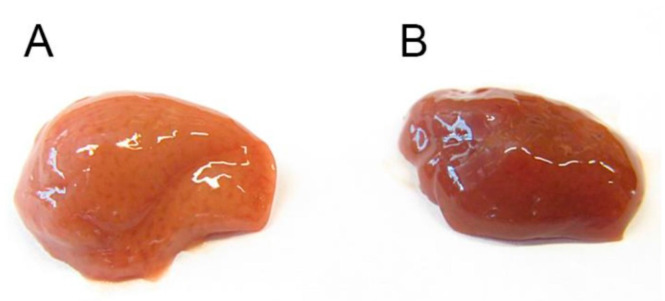
Morphological appearance of liver of (**A**) non-treated *db/db* mouse and (**B**) deh-T3β-treated *db/db* mouse liver.

Fat accumulation is the main characteristic of the diabetic *db/db* mouse liver, which appears as a pink-colored liver (Figure 14A) instead of the reddish color in normal healthy non-diabetic mice. We treated *db/db* mice with deh-T3β in the diet for 2 months and compared the livers of the non-treated *db/db* mice (Figure 14A) to those of treated *db/db* mice (Figure 14B). As shown in Figure 14B, the liver from the treated mice had a normalized appearance with a characteristic blue-red color.

### 3.15. Triglyceride Content in Mice Liver and Blood 

**Figure 15:**
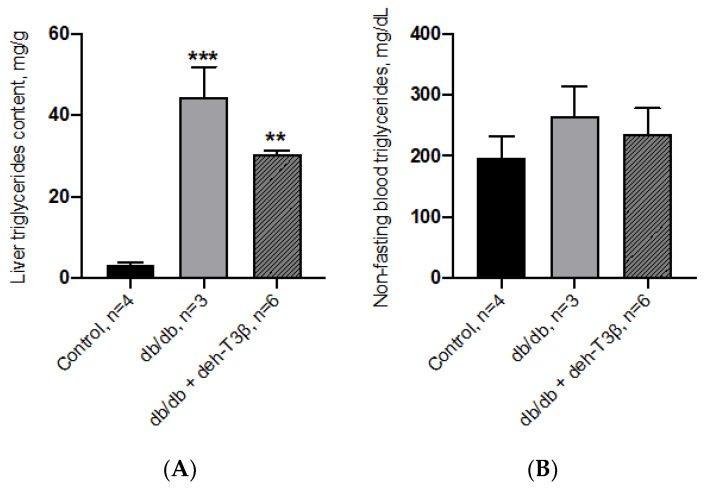
Triglyceride levels in the liver (**A**) and in plasma (**B**) of control C57BL6 mice and *db/db* mice non-treated or treated with deh-T3β in the diet for two months, ** *p* < 0.001, *** *p* < 0.001.

The triglyceride contents of the liver were analyzed in wt C57BL6, non-treated, and deh-T3β-treated *db/db* mice. The *db/db* mouse liver is characterized by a high content of fat, mainly triglycerides. A significant decrease (*p* = 0.03) was observed after two months’ oral treatment of the mice with deh-T3β mixed with the diet (Figure 15A). There were no significant differences in the plasma levels of triglycerides between the wt and *db/db* mice, non-treated and treated, but there was, a tendency towards differences despite the relatively small number of mice (Figure 15B).

The liver enzymes AST and ALT decreased by 50% in *db/db* mice treated with deh-T3β after one month compared to non-treated mice (not shown). However, treatment with deh-T3β did not affect the non-fasting plasma glucose levels, while the fasting levels decreased from a mean of 10 to 8 mmol/L in the *db/db* mice. The non-fasting levels were 20–30 mmol/L, independent of the treatment after one month. Body weight (40–50 g) was not different between the treated and non-treated *db/db* mice, while the fat/lean ratio was slightly reduced from 1.6 to 1.5, dependent on reduced adipose tissue in the deh-T3β-treated mice compared to the non-treated mice.

### 3.16. Adipokine Levels in Plasma of Deh-T3β Treated db/db Mice

**Figure 16:**
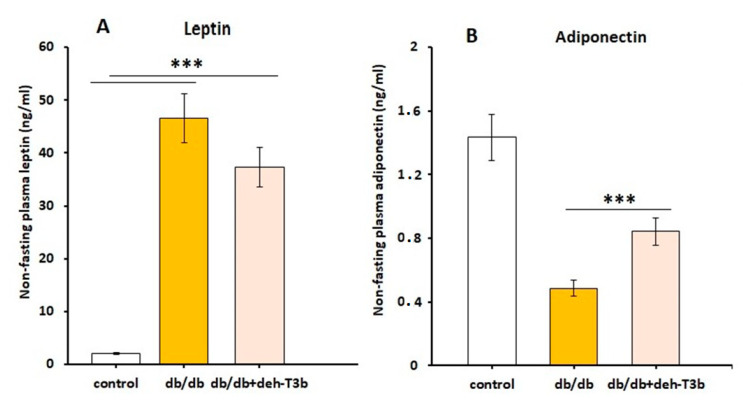
Leptin (**A**) and plasma levels of adiponectin (**B**) in control C57BL6 mice and *db/db* mice after two weeks’ treatment with chow or deh-T3β mixed with the diet. Values are means ± SEM of three independent experiments, *** *p* < 0.001.

Wt mice (n = 5) and *db/db* mice (n = 10) were treated for two months either with normal chow (wt (n = 5) and *db/db* (n = 5) controls) or with chow mixed with deh-T3β for *db/db* mice (n = 5). Leptin and adiponectin are adipokines produced by the adipose tissue. Leptin levels are extremely high in *db/db* mice due to the knockout of the leptin receptor. Thus, *db/db* mice develop obesity because of leptin resistance. When the mice were treated with deh-T3β, the leptin levels decreased slightly but not significantly (Figure 16A). Adiponectin levels were low in *db/db* mice compared to wt control mice. When treated with deh-T3β, the adiponectin levels of the *db/db* mice increased significantly (Figure 16B).

## 4. Discussion

In the present study of the *db/db* mouse type 2 diabetes model, we found that a newly synthesized derivate of tocotrienols has both antioxidant properties and beneficial effects on mitochondrial function. The compound improved mitochondrial function, which we think explains the improvement, despite persistent hyperglycemia, of many different late diabetes complications such as impaired wound healing, kidney dysfunction, fatty liver disease, and ischemic reperfusion injury of the heart. The glucose levels in the *db/db* mice were not affected by treatment with deh-T3β, which suggest that the positive effects on the heart, kidney, and liver were not due to normalization of glucose but possibly due to a decrease in free radical production and improved mitochondrial function.

The excessive production of ROS and reactive nitrogen species and insufficient removal of the free radicals will damage cellular proteins, lipids, and nucleic acids [21]. Oxidative stress occurs when ROS production exceeds the cellular antioxidant defense capacity. Hyperglycemia and obesity are some of the main factors involved in the excessive generation of ROS [22]. Free radicals and oxidative stress are involved in several pathological conditions such as diabetes complications, cancer, and Alzheimer’s disease [1,22]. During oxidative stress, cells respond by increasing the expression of antioxidants. Cells possess both enzymatic and non-enzymatic antioxidants. Coenzyme Q (CoQ) and vitamin E are two non-enzymatic antioxidants; the first is synthesized in all cells and the latter is acquired from the diet [1]. Tocotrienols are well-known antioxidants and members of the vitamin E family [23,24].

In a variety of conditions, such as cardiomyopathy, aging, muscle degeneration, cancer, and genetic disorders, tissue levels of CoQ are reduced, suggesting an impaired mitochondrial function [1]. In an effort to increase the biosynthesis of CoQ in cell culture system, HepG2 cells were cultured in the presence of tocotrienols and their modified (mono-and diepoxidated) forms. All naturally occurring tocotrienols and the epoxidated forms significantly increased CoQ biosynthesis in HepG2 cells [7]. We introduced several additional modifications into the side chains and into the chromanol structures of all four tocotrienols. One of them, mono-epoxy-tocotrienol-α, significantly enhanced both in vivo and in vitro wound healing in *db/db* mice and human fibroblast cells, respectively [8].

We introduced about fifty modifications and tested them in in vitro wound healing in HDF cells and in vitro angiogenesis in HDMVE cells. The modified compound that exerted a pronounced effect on the mRNA expression of the growth factors *PDGFB* and *VEGFA* and which enhanced in vitro wound healing in HDF cells and stimulated new tube formation in in vitro angiogenesis (in HDMVEC) was selected for further studies. Deh-T3β also stimulated the gene expression of *PDGFB* and *VEGFA* in the skin from orally *db/db* mice and was shown to have a significant wound healing effect in *db/db* mice.

Deh-T3β is a derivative of the naturally occurring β-tocotrienol (Figure 1) that was modified by introducing a double bond between positions three and four on the chromanol ring. Tocotrienols are known to possess potent antioxidant and anti-inflammatory capacities even superior to those of tocopherols [23]. Mechanistic and preclinical studies suggested that tocotrienols and their long-chain metabolites have stronger anti-inflammatory effects than their precursors [25]. The anti-inflammatory aspect of tocotrienols and their metabolites might be mediated by their radical scavenging properties. Like tocopherols, tocotrienols are metabolized through the oxidative degradation of their side chains catalyzed by cytochrome-P450 [26]. It is possible that deh-T3β and its metabolites separately or collectively contribute to the beneficiary effects we observed in this study. When H9C2 cells were incubated with deh-T3β with or without H_2_O_2_, improved cell viability and oxygen consumption rate were observed, demonstrating the protective and/or antioxidant property of deh-T3β. The metabolite distribution study showed that most of the methanol/water-soluble radioactive extract was found in the blood but also in the kidneys and the heart, which suggested that metabolites were taken up by the kidneys and the heart. The chloroform-soluble part of the metabolite and/or the intact form of deh-T3β was abundantly accumulated in the liver, skin, WAT, BAT, and abdominal fat of the *db/db* mice after 10 days’ treatment. This lipid-soluble compound, deh-T3β, was also accumulated in the heart. These findings may explain the effect on liver steatosis, heart function, wound healing, and the changes in the adipokines produced by the adipocytes.

The lipid-soluble deh-T3β is probably absorbed the same way as the other vitamin E isomers in the small intestine, assisted by bile salts, after which it is packaged in chylomicrons and lipoproteins and transported in the lymphatic system and circulation. Tocotrienols are taken up by the cells in the receptor-mediated endocytosis of lipoproteins, which may also involve lipoprotein digestion by lipases [27].

Reactive oxygen species are not only harmful but are also essential participants in multiple physiological signaling pathways and regulating biological processes, also in the heart [28]. There are multiple sources of ROS production in the heart, including those arising from mitochondria and cytochrome P450. When H9C2 cells were incubated with deh-T3β with or without H_2_O_2_, improved cell viability and oxygen consumption rate were observed, demonstrating the protective effect of deh-T3β on mitochondrial function. Additionally, other proteins such as p66 and p47 are known to trigger mitochondrial oxidative stress by accepting electrons from cytochrome C, resulting in the formation of H_2_O_2_ [18,19]. We investigated the expression of these proteins in the heart by Western blot, and the expression of both p66 and p47 in the deh-T3β-treated mice heart decreased significantly compared to the non-treated *db/db* mice. Type 2 diabetes is one of the leading causes of the development of myocardial infarction. Cardiovascular complications, including ischemic heart disease, in type 2 diabetes are caused by increased oxidative stress and reduced bioavailability of nitric oxide (NO) because of reduced production of endothelial nitric oxide synthase (eNOS), caused by excessive ROS production [29]. It was shown that arginase is an important regulator of NO production, which competes with eNOS for their common substrate L-arginine. Increased arginase activity is an important regulator of NO formation and ROS production in diabetes, and it is upregulated in the RBCs of diabetes patients [13]. Studies in recent years suggested that RBCs are involved in several vital cellular functions including redox balance regulation and release of bioactive NO and ATP, which are involved in tissue protection and regulation of cardiovascular homeostasis [30]. We therefore compared RBCs from *db/db* mice treated with deh-T3β and RBCs from non-treated *db/db* mice on the heart from wild-type mice for recovery after reperfusion injury. A significant (60%) recovery effect was observed in the heart when RBCs from deh-T3β treated mice were used, indicating the effectiveness of the product in suppressing oxidative stress. It is plausible that deh-T3β exerts its beneficial effects in the heart both as an antioxidant reducing ROS in RBCs and through a direct effect, inhibiting arginase activity and thereby stimulating eNOS activity. Previous studies also showed that other antioxidants inhibit arginase activity [30].

The short-term effects of deh-T3β include changes in gene expression, which may cause a cascade of events, stimulating signaling pathways including reduction of oxidative stress and improvement in mitochondrial function. In the long term, this will prevent late diabetes complications. We previously found similar effects with CoQ10 treatment despite chronic hyperglycemia in *db/db* mice preventing nephropathy and neuropathy after several months of treatment [31]. Diabetic nephropathy is associated with mitochondrial dysfunction and ROS production. The mitochondrial permeability transition pore that traverses both the outer and inner mitochondrial membranes is involved in the restoration of the ionic balance between the matrix and the cytosol [32]. Persistent opening of this pore during diabetic conditions results in mitochondrial swelling, which, in the absence of appropriate treatment, can lead to cell death. eh-T3β treatment of *db/db* mice showed a substantial decrease in kidney mitochondrial swelling compared to the non-treated mice. Additionally, a substantial recovery of both glomerular and tubular structures of the kidney and decreased levels of creatinine in the plasma were observed upon treatment with deh-T3β compared to non-treated *db/db* mice. We previously reported that treatment with CoQ in *db/db* mice improved kidney function with improved mitochondrial function [33].

The *db/db* mice are characterized by obesity, dyslipidemia and fatty liver. Morphological appearance of the liver after treatment with deh-T3β showed substantial improvement in the fatty liver. Triglyceride levels the liver was also significantly reduced after treatment of *db/db* mice with deh-T3β despite no significant weight loss. Increased levels of serum alanine transferase (ALT) and aspartate transferase concentrations which correlates positively with fatty liver and inflammation, decreased in the deh-T3β-treated *db/db* mice compared to those non-treated *db/db* mice.

The decreased levels of leptin suggest a reduced amount of adipose tissue; however, this was very moderate. We cannot exclude that deh-T3β may have a direct effect on leptin synthesis. Increased levels of adiponectin suggest reduced inflammation in the adipose tissue. A combination of the changes in leptin and adiponectin suggest an improved insulin sensitivity primarily in adipose tissue [34]. Increased adiponectin production implies an activation of AMPK and increased protection against cell stress. Increased production of oxidants in the mitochondria causes insulin resistance and is associated with low levels of the antioxidant coenzyme Q in the insulin-resistant fat and muscle tissue mitochondria of mice [35]. Supplementation with CoQ restored normal oxidant levels and prevented the development of insulin resistance [36]. Compounds that induce the biosynthesis of CoQ or increase CoQ in mitochondria, as well as compounds with antioxidant effects, may prevent or reverse insulin resistance.

Diabetes mellitus is one of the main reasons for impaired wound healing. There are multiple pathogenic factors explaining this delayed wound healing, including hyperglycemia, hypoxia, impaired HIF-1α activity, and the impaired expression and activities of the cytokines and growth factors required for normal healing process [37]. Previously, we showed that HIF1α, carnosine, and epoxidated α-tocotrienol, all of which are competent antioxidants, effectively enhanced wound healing in *db/db* mice [8,12,38]. It was previously shown that antioxidants such as CoQ10 normalized hyperglycemia induced inhibition of fibroblast proliferation in in vitro systems [39]. These findings suggest that the antioxidant properties of deh-T3β, including its angiogenic effects, explain the wound healing benefits we observed in this and in previous studies conducted with modified tocotrienols [8].

Microarray studies showed that the expression of the early response gene *NR4A1* increased by several folds. NR4A1 is a nuclear transcription factor that can be induced by several factors such as cytokines, glucose, fatty acids, exercise, and growth factors. It is associated with various physiological and pathological processes, such as metabolic disease, insulin synthesis, cardiovascular disease, inflammation, and carcinogenesis. Several naturally occurring compounds were identified as agonists that bind to NR4A1 and stimulate its transcriptional activities [40]. NR4A1 is indirectly associated with the NF-κB, PI3K, P38-MAPK, and MMP-9 signaling systems, which are all involved in cell stress, inflammation and cell proliferation. It is possible that deh-T3β or its metabolites may function as a NR4A1 agonists. This, however, needs further investigation.

Diabetic complications are caused by several mechanisms, among which oxidative stress, excess of free radicals, and mitochondrial dysfunction are central [41]. In this study, we examined the effect of deh-T3β on free radicals and mitochondrial function in relation to some diabetes complications. The microarray study suggested an early effect in the liver on some pathways in the nucleus, cytoplasm, and cell membranes. The strongest effect was observed in the nucleus, upregulating *NR4A1*, which is important for many central signaling pathways.

## 5. Conclusions

We found that deh-T3β exerts positive effects in several organs of diabetic mice without reducing non-fasting blood glucose levels. This suggests that deh-T3β has effects on downstream hyperglycemia at the cellular level and that both its antioxidant properties and improvement in mitochondrial function are involved, which is central to most cells. Further studies are required to elucidate the precise mechanisms by which deh-T3β exerts its beneficial effects at the molecular level.

## Data Availability

The data presented in this study are available on request from the corresponding author.
